# The Effect of Sanggua Drink Extract on Insulin Resistance through the PI3K/AKT Signaling Pathway

**DOI:** 10.1155/2018/9407945

**Published:** 2018-02-19

**Authors:** Yu Cai, Ying Wang, Fei Zhi, Qi-Chang Xing, Yun-Zhong Chen

**Affiliations:** ^1^College of Pharmacy, Hubei University of Chinese Medicine, Wuhan 430065, China; ^2^Institute of Engineering Technology of Chinese Traditional Medicine and Health Food of Hubei Province, Wuhan 430065, China

## Abstract

Treating type 2 diabetes mellitus (T2DM) using thiazolidinediones and biguanides can present several challenges for patients. Sanggua Drink (SGD) is a commonly used agent in traditional Chinese medicine, and it consists of* Folium Mori*,* Fructus Momordicae Charantiae*,* Radix Puerariae Lobatae*, and* Rhizoma Dioscorea*. The hypoglycemic effects and mechanisms of SGD extracts on insulin resistance in diabetic rats were investigated. SGD (1.24 g/kg orally) was verified in T2DM rats induced by a high-fat diet and streptozotocin. The results showed that SGD treatment was observed to reduce fasting blood glucose, water and food intake, total cholesterol triglycerides, and LDL, OGTT, FINS, HOMA-IR, GHb, and MDA and increase hepatic glycogen, HDL, SOD, CAT, and GSH-Px in diabetic rats. Simultaneously, SGD treatment by T2DM showed significantly ameliorated pathological changes and reduced inflammation in the pancreas. Treatment was also observed to increase gene and protein expressions of InsR, IRS-2, PI3K, AKT, and Glut4 in the livers of diabetic treated rats. These results suggest that SGD extracts have hypoglycemic properties and may alleviate insulin resistance in T2DM rats through the PI3K/AKT pathway. Therefore, SGD appears to be a promising insulin sensitizer.

## 1. Introduction

Type 2 diabetes mellitus (T2DM) is a metabolic syndrome with an increasing prevalence worldwide. To date, there are an estimated 37.1 million T2DM patients worldwide. Among them, 11.6% and 11.3% are found in China and the United States, respectively [1, 2]. The main etiology of T2DM is insulin resistance (IR), which causes cells to stop responding adequately to the standard actions of insulin. While the body may continue to produce insulin, the cells in the body become resistant to its actions. As such, cells cannot process it effectively, which can lead to hyperglycemia [3]. To reduce IR, several insulin sensitizers are commonly used, including thiazolidinediones (e.g., rosiglitazone and pioglitazone) and biguanides (e.g., metformin). These agents can improve IR and increase the peripheral utilization of insulin, which results in a decrease in blood glucose.

Thiazolidinediones are associated with several risks, including bone fractures, bladder cancer, and, of special concern, cardiovascular side effects [4]. Some studies have shown that thiazolidinediones carry no additional risks for myocardial infarction or cardiac death [5, 6]. However, the U.S. Food and Drug Administration has recommended that they should be used with caution. The application of thiazolidinediones is restricted in some countries due to some safety issues.

Although biguanides may have gastrointestinal side effects, they are frequently used in the treatment of IR. However, some T2DM patients, especially those who cannot tolerate biguanides, encounter difficulties using insulin sensitizers in a clinical setting. Therefore, new research is necessary to develop antidiabetic agents that sustain equivalent therapeutic effectiveness but with fewer side effects [7].

SGD consists of* Folium Mori, Fructus Momordicae Charantiae, Radix Puerariae Lobatae*, and* Rhizoma Dioscoreae*, all of which were provided by Professor Jiageng Li of Hubei University of Chinese Medicine [8]. These ingredients nourish yin, tonify the liver and kidneys, clear heat, and moisten dryness. They have been officially classified according to the homology of medicine and food by the Ministry of Health. Meanwhile, several studies have confirmed that these four plants possess hypoglycemic effects [9–18]. However, few studies have assessed the possible hypoglycemic properties of SGD, and no experimental evidence exists to prove its hypoglycemic effect.

This study aims to determine the hypoglycemic effects of SGD extracts by investigating metabolic, biochemical, and molecular parameters. Body weight, food and water consumption, fasting glycemia, blood triglycerides, total cholesterol and protein, insulin, hepatic glycogen, and protein expression in the insulin signaling pathway of livers in streptozotocin-diabetic rats were investigated.

## 2. Materials and Methods

### 2.1. Plant Material and Extraction

*Folium Mori*,* Radix Puerariae Lobatae*, and* Rhizoma Dioscoreae* were purchased from Hubei University of Chinese Medicine, and* Fructus Momordicae Charantiae* was collected from Jiaxiang District, Wuhan City. All plants were authenticated by Dr. Zhigang Hu at the drug research office of Hubei University and combined together according to a prescription ratio. The procedure of preparing the SGD extract was according to the method by Wang et al. [19].

### 2.2. Animals

SPF male SD rats aged 2 months and weighing 190 ± 10 g were obtained from Hubei Provincial Center for Disease Control and Prevention. The animal license number was SYXK (Hubei) 2012-0068. The experimental animal production license number was SCXK (Hubei) 2015-0018. The rats were housed under normal environmental conditions (22 ± 2°C) with a 12-hour light/dark cycle. Food and water were available ad libitum. Rats were randomly divided into two groups and fed with either a high-fat diet (68% basic feed, 15% sugar, 10% ripe lard, 5% egg yolk powder, 1% cholesterol, and 1% sodium cholate) as the experimental group or a normal diet as the control group. The rats were sacrificed by CO_2_ exposure and decapitated thereafter. Experimental protocols were approved by the Animal Experimentation Ethics Committee of Hubei University of Chinese Medicine.

### 2.3. Induction of Experimental Diabetes

At the 28th day, an injection of 40 mg/Kg streptozotocin (STZ, Sigma, St. Louis, MO) was administered to the rats to induce diabetes after a 12-hour starvation period. The STZ was dissolved in a citrate buffer (pH 4.5) and directly administered intraperitoneally. The rats were starved for three hours after injection and were then given a 3% glucose solution to prevent hypoglycemia. After a three-day period, the rats were fasted again for 10 to 12 hours and blood glucose was quantified using a glucometer (Beijing Yicheng Biology Electronic Technology Co., Ltd.). Those rats whose fasting glucose was between 11.1 and 33.3 mmol/L and stayed stable for two weeks were considered diabetic and were included in this study.

### 2.4. Treatment

The rats were separated at random into the following four groups (*n* = 8/group): control group (nondiabetic rats treated with saline), diabetic group (diabetic rats treated with saline), metformin group (diabetic rats treated with metformin), and SGD group (diabetic rats treated with SGD extract). Saline at a dosage of 3 ml/kg b.w., metformin at a dosage of 150 mg/kg b.w., and an extract of SGD at a dose of 1240 mg/kg b.w. were dissolved in saline and administered orally by gavage daily for 42 successive days.

### 2.5. Fasting Blood Glucose, Body Weight, and Food and Water Intake

Body weight and food and water intake were observed daily. After completing the study, the rats were sacrificed and blood samples were recovered and centrifuged to acquire the serum. Fasting blood glucose was measured on a weekly basis using a glucometer (Beijing Yicheng Biology Electronic Technology Co., Ltd.).

### 2.6. Oral Glucose Tolerance Test, Fasting Insulin, and HOMA-IR

Oral glucose tolerance tests (OGTT) were conducted, and blood from the tail vein was collected at 0, 30, 60, and 90 minutes after glucose lavage (at 1 g/kg of body weight), following a 12-hour overnight fast. Blood glucose was measured using a glucose meter (Beijing Yicheng Biology Electronic Technology Co., Ltd.), and insulin was detected by enzyme-linked immunoassay (ELISA; rat insulin ELISA kit, Hualian Branch Biotechnology Co., Ltd.) according to the manufacturer's instructions. A homeostatic model assessment of insulin resistance (HOMA-IR) was calculated as follows [17]:

(1) $$\begin{array}{c} HOMA\text{-}IR=\frac{FBG\left(mmol/L\right)\times FINS\left(\mu U/mL\right)}{22.5}, \end{array}$$

where FBG is fasting blood glucose and FINS is fasting insulin concentration.

### 2.7. Total Cholesterol, Triglycerides, High-Density Lipoprotein Cholesterol, and Low-Density Lipoprotein Cholesterol

Total cholesterol (TC), triglycerides (TG), high-density lipoprotein cholesterol (HDL-C), and low-density lipoprotein cholesterol (LDL-C) were measured by commercial kits (Nanjing Jiancheng Institute of Bioengineering, China).

### 2.8. Hepatic Glycogen, Glycosylated Hemoglobin, SOD, MDA, CAT, and GSH-Px

Hepatic glycogen content was measured using the following method. Firstly, fresh liver (⩽100 mg) samples were collected and washed with saline according to the sample weight (mg) : lye volume (ul) = 1 : 3. Secondly, the samples were bathed in boiling water for 20 minutes and cooled with flowing water. Thirdly, 1% glycogen solution was prepared and detected at 620 nm. Finally, the contents of glycosylated hemoglobin (GHb), SOD, MDA, CAT, and GSH-Px were quantified using kits (Nanjing Jiancheng Institute of Bioengineering, China) in the Synergy 2 Multifunctional Microplate Reader (BioTek Instruments, Inc., USA) according to the manufacturer's instructions.

### 2.9. Morphology and Histopathology Analysis

Rats were sacrificed according to international guidelines, and their pancreas tissues were removed and macroscopically examined. The tissues were fixed in a 10% formalin solution and dehydrated for 1 hour using a 30–100% series of alcohol and xylene and were later embedded in wax at 60°C. Paraffin blocks containing the tissues were divided into sections of 5*μ*m thickness. The sections were stained with hematoxylin and eosin for morphological assessment. The samples were double-blindly assessed and reviewed by two pathologists under a microscope at ×200 magnification.

### 2.10. Reverse Transcription-PCR Analysis

Total RNA contained within the rat livers was collected using the TRIzol method according to the manufacturer's instructions. The gene expressions of InsR, IRS-2, PI3K, Akt, Glut-4, and *β*-actin were distinguished by reverse transcription-polymerase chain reaction (RT-PCR) analysis. Primer sequences and PCR conditions are listed in Table 1. Gene expression relative quantification results were normalized on the level of *β*-actin transcripts. The results are expressed as the average of three independent experiments in triplicate.

### 2.11. Protein Expression by Western Blotting

The rat livers were acquired and homogenized in an ice-cold cell lysis buffer (Wuhan Servicebio Technology Co., Ltd.). Protein concentrations were quantified using the BCA method according to the manufacturer's instructions (Wuhan Servicebio Technology Co., Ltd.). The sample was chromatographed in a polyacrylamide gel with appropriate pores (Beijing Liuyi Instrument Factory) for each molecular weight. After electrophoresis, the protein was transported to a nitrocellulose membrane (Millipore) at a constant voltage of 120 with 20% methanol and 0.02% SDS. The protein was immediately treated with 5% BSA base solution (Roche) overnight and washed with a base solution at room temperature. The protein was detected in the membrane after a three-hour incubation period at 4°C using a primary antibody (Wuhan Servicebio Technology Co., Ltd.) in each of the basic solutions containing 3% albumin. The protein was then washed and incubated using a peroxidase-conjugated secondary antibody-HRP solution containing basal solution and 1% skimmed milk at room temperature. The membranes were washed and incubated in the darkness with a luminol chemiluminescent substrate (Pierce, Rockford, IL) and exposed to an autoradiographic film (Kodak T-MatG/RA, Rochester, NY). The intensities of the bands were measured by densitometry (Epson Expression 1600, Long Beach, CA). The membrane was washed and incubated with a luminol chemiluminescent substrate (Wuhan Servicebio Technology Co., Ltd.) in a dark chamber and exposed to a radiation self-developed film (Wuhan Servicebio Technology Co., Ltd.). The optical density of the target band was analyzed by software (Alpha Innotech).

### 2.12. Statistical Analysis

The data were expressed as mean ± standard deviation. For multiple comparisons, one-way analysis of variance (ANOVA) was used, followed by Tukey's test. For comparisons between two groups, Student's *t*-test was used. A value of *P* < 0.05 was considered statistically significant. Experimental data was processed using IBM SPSS Statistics 19 statistical software.

## 3. Results

### 3.1. Effect of SGD Treatment on Body Weight and Food and Water Intake

As of the 42nd day of treatment, the body weight of the diabetic rats had markedly decreased compared with the body weight of rats in the control group (Table 2) (*P* < 0.01). No marked differences were discovered between the body weights of diabetic rats in the STZ and SGD groups. Diabetic rats were observed to drink more water compared to rats in the control group (Table 3) (*P* < 0.01). A decrease of 42% in water consumption was observed in rats in the SGD group compared to diabetic rats that were given saline (*P* < 0.05). No differences between the positive and SGD groups were observed. A significant increase in food consumption was observed in diabetic rats compared with rats in the control group (Table 4) (*P* < 0.01). No difference between positive control and SGD groups was observed.

### 3.2. Effect of SGD Treatment on Fasting Blood Glucose

A significant increase was observed in blood glucose in the diabetic group compared with the control group (*P* < 0.01). At the end of the treatment, a marked decrease was observed in blood glucose in the SGD compared with the diabetic group (*P* < 0.01). A decrease of 40% was shown in blood glucose in SGD group rats compared with rats in the diabetic group. No difference in blood glucose was discovered between positive and SGD groups (Table 5).

### 3.3. Effect of SGD Treatment on OGTT, FINS, and HOMA-IR

**Figure 1:**
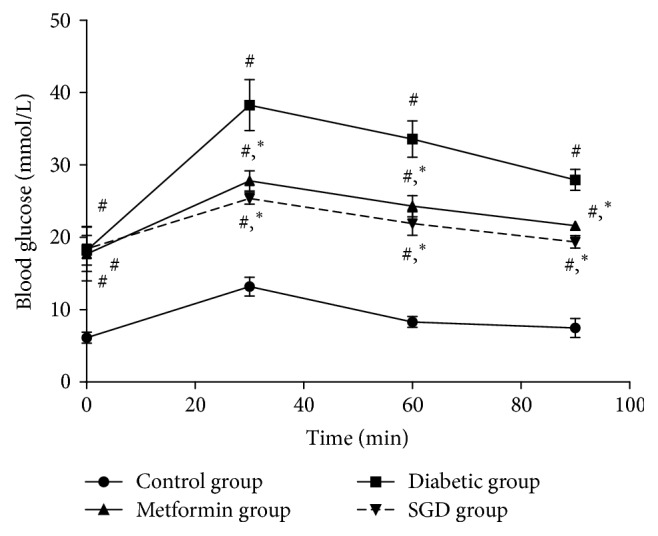
OGTT at 42 days. Values are expressed as mean ± SD. ^#^*P* < 0.05 versus the control group; ^*∗*^*P* < 0.05 versus the diabetic group.

For the OGTT test, the diabetic rats showed a significant increase in glycemia at 0, 30, 60, and 90 minutes (*P* < 0.05) after glucose administration (1 g/Kg) compared with rats in the control group. A marked decrease in blood glucose was found in rats given the SGD extract at 30, 60, and 90 minutes (*P* < 0.05) compared with the diabetic group (Figure 1). No difference in blood glucose was observed between rats in the positive and SGD groups. For the insulin test, insulin levels in the diabetic group were higher than the levels of rats in the control group (*P* < 0.01). However, the level of insulin in the SGD-treated group was observed to decrease markedly compared with the untreated diabetic group (*P* < 0.01, Table 6). No difference in insulin levels between the SGD and metformin group was observed. To evaluate IR, HOMA-IR was calculated by the formula described above. After comparisons, the HOMA-IR value of the diabetic group was observed to significantly improve compared to the control group, while the HOMA-IR value in the SGD-treated group was markedly reduced compared with the untreated diabetic group (*P* < 0.01, Table 6). No difference in HOMA-IR was discovered between the SGD and the metformin groups. Therefore, it is reasonable to suggest that SGD can reduce IR in treated diabetic rats.

### 3.4. Biochemical Parameters

Total triglycerides (TG), cholesterol (TC), and low-density lipoprotein cholesterol (LDL-C) were shown to improve in diabetic rats, while high-density lipoprotein cholesterol (HDL-C) was observed to decrease compared with the control group (*P* < 0.01, Table 7). SGD treatment over 42 days was observed to reduce TG, TC, and LDL-C levels and increase HDL-C levels in rats in the SGD group compared with the STZ group, respectively (*P* < 0.05 or *P* < 0.01). A decrease of 46.8%, 14.9%, and 22.8% in the levels of TG, TC, and LDL-C was shown in the SGD group compared to the diabetic group, respectively. An increase of 37.1% was observed in the levels of HDL-C in the SGD group compared to the diabetic group. GHb levels were observed to increase in the STZ group compared with the control group (*P* < 0.01, Table 7). Decreases of 16.1% and 16.3% in the content of GHb were observed in the positive and SGD groups compared to the diabetic group, respectively (*P* < 0.05). No difference in GHb between the positive and SGD groups was observed. The level of hepatic glycogen was observed to decrease to 27.5% in the STZ group compared with the control group (*P* < 0.01). Increases of 28.1% and 36.4% were observed in the content of hepatic glycogen in the positive and SGD groups compared with the diabetic group, respectively (*P* < 0.05). No difference in hepatic glycogen was observed between positive and SGD groups.

### 3.5. Effect of SGD Treatment on SOD, MDA, CAT, and GSH-Px

SOD, CAT, and GSH-Px were decreased in diabetic rats, while MDA was increased compared with the control group (*P* < 0.01). SGD treatment over 42 days was observed to increase SOD, CAT, and GSH-Px levels and decrease HDL-C levels in rats in the SGD group when compared with rats in the STZ group, respectively (*P* < 0.05 or *P* < 0.01). Increases of 6.8%, 132.2%, and 51.1% in the levels of SOD, CAT, and GSH-Px in the SGD group were observed in the diabetic group, respectively. A decrease of 37.0% was observed in the levels of MDA in the SGD group compared with the diabetic group. No difference in SOD, CAT, GSH-Px, and MDA was observed between the positive and SGD groups (Table 8).

### 3.6. Effect of SGD Treatment on Morphology

**Figure 2:**
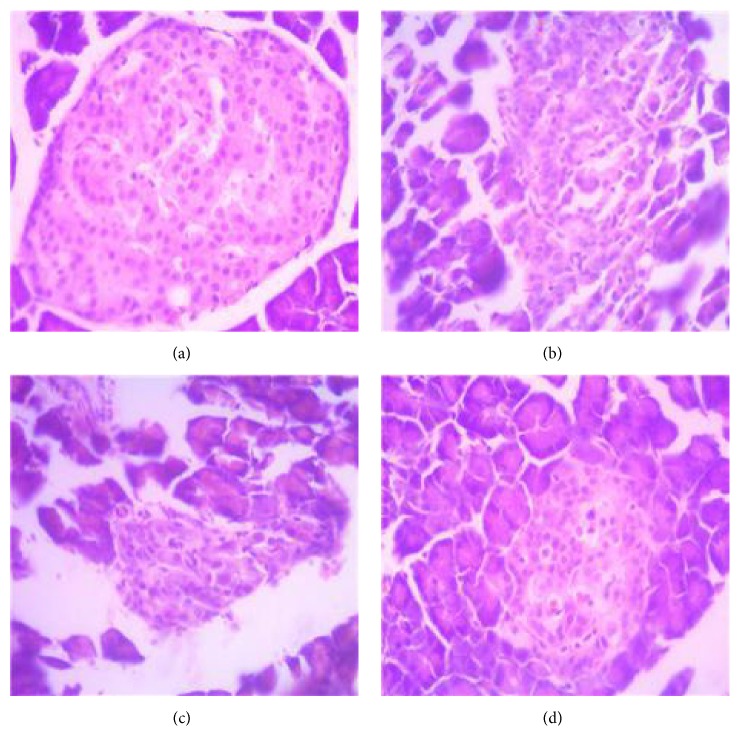
The pictures of H&E staining. The pancreas tissues of rats were stained with hematoxylin and eosin and examined under a microscope at ×200 magnification. (a) Control group. (b) Diabetic group. (c) Metformin group. (d) SGD group.

The structure of pancreatic islets in the normal group was clear and normal (Figure 2). In the diabetic group, no obvious islet cell boundary was observed, and cell arrangement was disordered. In the metformin group, the arrangement of islet cells was disordered, and inflammatory cells were observed to infiltrate. No inflammatory cells or less inflammatory cells in the SGD group were observed, which was similar to the normal group.

### 3.7. Reverse Transcription-Polymerase Chain Reaction Analysis

The PI3K/AKT signaling pathway is considered important in IR. The rat livers were removed to detect the effect of SGD on IR. PI3K/AKT signaling pathway-related gene expression levels were detected by RT-PCR analysis, including InsR, IRS-2, PI3K, Akt, and Glut-4 genes. The primers used are listed in Table 1. All gene expression levels were observed to significantly reduce in the diabetic group compared with the control group (*P* < 0.05). After SGD treatment, gene expression levels were observed to improve compared with the diabetic group (*P* < 0.05, Table 9).

### 3.8. Protein Expression by Western Blotting

**Figure 3:**
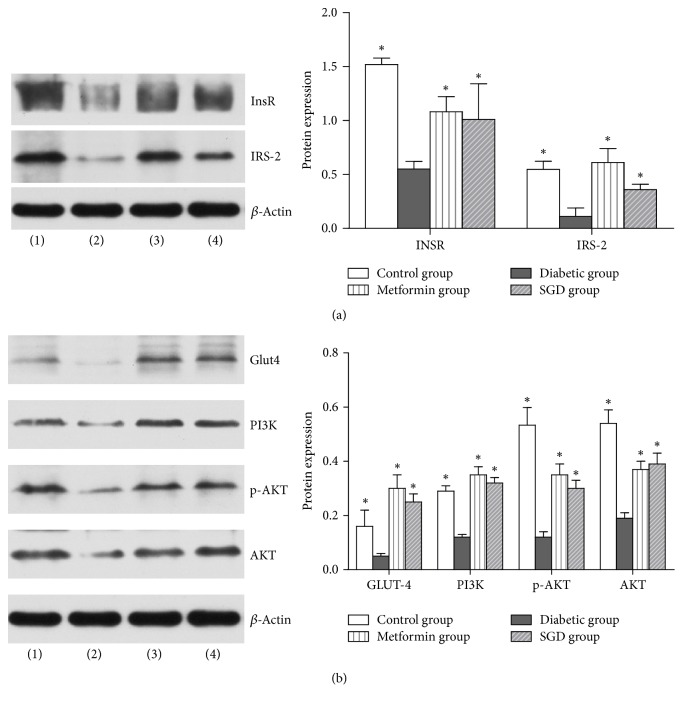
Protein expression in the rat liver. Western blot of InsR and IRS-2 (a) and Glut4, PI3K, p-AKT, and AKT (b). (1) Control group. (2) Diabetic group. (3) Metformin group. (4) SGD group. Values are expressed as mean ± SD. ^*∗*^*P* < 0.05 versus the diabetic group.

InsR, IRS-2, PI3K, Akt, p-Akt, and Glut-4 expression levels were measured in the rat livers in all groups. The relative expression of InsR, IRS-2, PI3K, Akt, p-Akt, and Glut-4 showed a significant increase in the SGD group compared with rats in the diabetic group (*P* < 0.05). No difference between the SGD group and the metformin group was observed. All results are shown in Figure 3.

## 4. Discussion

T2DM is an increasingly common medical condition that threatens nearly 1 in 10 people worldwide. A trend for an increasing prevalence is observed every year. Therefore, the question of how to best target T2DM is an important issue for investigators worldwide.

In this study, we investigated the hypoglycemic activity and effects of SGD extracts which were administered in a high-fat diet to streptozotocin-induced diabetic rats. Hyperglycemia resulting from DM is a consequence of defects or a lack of insulin secretion and/or action [20]. Several experimental models of diabetes involve using rodents to replicate the symptoms of diabetes. Alloxan and streptozotocin are commonly administered to induce diabetes in rodents [21]. The symptoms of animals with STZ-induced diabetes include polyphagia, polydipsia, polyuria, and weight loss, all of which were observed in the streptozotocin-injected rats in the present study. The dose of SGD which was administered to rats was changed according to human adult dose, and we observed no side effects during the whole process of the experiment. Water intake was observed to decrease in rats in the SGD group compared to rats in the diabetic group. Body weight and food intake were observed to decrease in diabetic rats, and treatment with SGD was not observed to affect weight loss and food intake. After 42 days of treatment with SGD extracts, hyperglycemia was observed to significantly decrease in rats in the SGD group compared with diabetic rats. The decrease began on the 28th day of treatment and continued until study termination, thus demonstrating the hypoglycemic effect of the treatment. The OGTT showed a significant increase in glucose tolerance of SGD-treated animals when compared with diabetic rats. The levels of insulin, TG, TC, LDL-C, GHb, and MDA, as well as HOMA-IR values, were all observed to significantly decrease in the SGD-treated group compared with the diabetic group. The contents of HDL-C, SOD, CAT, GSH-Px, and hepatic glycogen were observed to significantly increase in the SGD-treated group compared with the diabetic group. Like the normal group, no or less inflammatory cells were observed in the SGD group. These results are in accord with the literature on the hypoglycemic effect of* Folium Mori*,* Momordica Charantia*,* Radix Puerariae Lobatae*, and* Rhizoma Dioscoreae *[9–18]. The relationship between insulin resistance and obesity was extensively studied. Chronic energy excess and consequent obesity may cause a serious problem: insulin resistance. When large amounts of glucose and saturated fat enter the cells from the blood flow, this may cause hypoxia, inflammation, and macrophage infiltration of adipose tissue, which may lead to oxidative stress and insulin resistance. An excessive amount of saturated fatty acids accumulates and circulates in insulin-sensitive tissues, disturbing the function of insulin. In this case, excessive lipid accumulation in the liver may play a primary and pathogenic role in insulin resistance [22]. Therefore, it is reasonable to suggest that SGD can decrease both blood glucose and IR in diabetes.

The PI3K/AKT signaling pathway is a classic insulin pathway because it plays a role in glucose uptake by the liver, skeletal muscles, and adipose tissues [23, 24]. Decreasing or blocking this pathway can reduce the physiological effects of insulin, which may lead to IR. Insulin combines with InsR on the surface of hepatocytes. The inner-membrane portion of InsR has a tyrosine kinase domain that phosphorylates IRS-2. IRS-2 plays a role in multiple pathways in the liver, in this case activating PI3K, which causes Akt to translocate from the cytoplasm to the cytomembrane. There, Akt is directly or indirectly activated by PDK1. Activated Akt transmits the signal to the downstream receptor substrate and produces various biological effects [25–27], such as glucose absorption, glycolysis, glycogen synthesis, and protein synthesis. To further investigate the mechanisms of SGD on IR, RT-PCR and western blot were used to test the PI3K/AKT signaling pathway-related genes in the liver, including InsR, IRS-2, PI3K, AKT, and Glut4. All gene expression levels were significantly decreased in diabetic rats. After SGD treatment, gene expression levels were increased, which indicates alleviators of IR in all insulin-affected tissues. After SGD treatment, increased InsR, IRS-2, PI3K, AKT, p-AKT, and Glut-4 expressions were observed, which accords with the regulation of gene expression. Therefore, it is reasonable to suggest that SGD may improve IR through the PI3K/AKT pathway.

Given the issues associated with the clinical use of insulin sensitizers, a dilemma may exist regarding the choice of treatment. The present investigation shows the anti-IR effects of SGD through the PI3K/AKT pathway and provides evidence for its application. Finally, it is reasonable to assume that SGD may be promising for the treatment of T2DM in clinical medicine.

## Figures and Tables

**Table 1:** Primers of reverse transcription-PCR analysis for genes.

	Primer sequence	Product length (bp)
R-Insr-S	CAATGGTGCTGAGGACACTAGG	139
R-Insr-A	GTGCTCTTCGTGGCTTGTGG	
R-IRS-2-S	TGACCAGTCCCACATCAGGC	123
R-IRS-2-A	CTGCACGGATGACCTTAGCG	
R-Glut4-S	CTGTTGCCCTTCTGTCCTGA	147
R-Glut4-A	CAACTTCCGTTTCTCATCCTTCA	
R-PI3K-S	GGTGAGGAACGAAGAATGGC	176
R-PI3K-A	TCCGAGGCAAGACAGGGATA	
R-AKT(rz)-S	TGAGACCGACACCAGGTATTTTG	135
R-AKT(rz)-A	GCTGAGTAGGAGAACTGGGGAAA	
R-*β*-actin-S	TGCTATGTTGCCCTAGACTTCG	240
R-*β*-actin-A	GTTGGCATAGAGGTCTTTACGG	

**Table 2:** Effect of the administration of saline (3 mL/Kg), metformin (150 mg/Kg), or SGD (1240 mg/Kg) on body weight (g) of male rats.

	0 days	14 days	28 days	42 days
Control group	380 ± 16^*∗∗*^	450 ± 21^*∗∗*^	510 ± 24^*∗∗*^	520 ± 25^*∗∗*^
Diabetic group	350 ± 13	325 ± 12	320 ± 14	300 ± 15
Metformin group	350 ± 14	340 ± 11	330 ± 12	320 ± 16
SGD group	340 ± 12	300 ± 9	320 ± 10	300 ± 15

Values are expressed as mean ± SD. ^*∗∗*^*P* < 0.01 versus the diabetic group.

**Table 3:** Effect of the administration of saline (3 mL/Kg), metformin (150 mg/Kg), or SGD (1240 mg/Kg) on water intake (ml/kg body weight/day) by male rats.

	0 days	14 days	28 days	42 days
Control group	152 ± 11^*∗∗*^	155 ± 13^*∗∗*^	153 ± 17^*∗∗*^	151 ± 20^*∗∗*^
Diabetic group	1047 ± 19	1212 ± 23	1223 ± 21	1257 ± 19
Metformin group	1024 ± 15	1007 ± 9	994 ± 25	912 ± 11^*∗*^
SGD group	1105 ± 21	1003 ± 11	1001 ± 18	954 ± 122^*∗*^

Values are expressed as mean ± SD. ^*∗*^*P* < 0.05 versus the diabetic group; ^*∗∗*^*P* < 0.01 versus the diabetic group.

**Table 4:** Effect of the administration of saline (3 mL/Kg), metformin (150 mg/Kg), or SGD (1240 mg/Kg) on food intake (g/kg body weight/day) by male rats.

	0 days	14 days	28 days	42 days
Control group	161 ± 11^*∗∗*^	160 ± 11^*∗∗*^	140 ± 16^*∗∗*^	125 ± 9^*∗∗*^
Diabetic group	346 ± 15	320 ± 17	320 ± 20	325 ± 21
Metformin group	309 ± 16	270 ± 19	260 ± 18	255 ± 23
SGD group	315 ± 13	310 ± 16	310 ± 15	300 ± 19

Values are expressed as mean ± SD. ^*∗∗*^*P* < 0.01 versus the diabetic group.

**Table 5:** Effect of the administration of saline (3 mL/Kg), metformin (150 mg/Kg), or SGD (1240 mg/Kg) on fasting blood glucose (mmol/L) of male rats.

	0 days	14 days	28 days	42 days
Control group	6.2 ± 0.6	6.1 ± 0.6	5.5 ± 0.3	5.7 ± 0.7
Diabetic group	18.2 ± 1.9^##^	20.4 ± 4.6^##^	22.5 ± 5.8^##^	24.2 ± 3.5^##^
Metformin group	17.6 ± 3.7^##^	16.2 ± 4.2	13.6 ± 4.2	12.3 ± 3.2^*∗∗*^
SGD group	18.4 ± 3.4^##^	16.9 ± 4.4	15.3 ± 5.7	14.5 ± 2.8^*∗∗*^

Values are expressed as mean ± SD. ^##^*P* < 0.01 versus the control group; ^*∗∗*^*P* < 0.01 versus the diabetic group.

**Table 6:** Effect of the administration of saline (3 mL/Kg), metformin (150 mg/Kg), or SGD (1240 mg/Kg) on FINS and HOMA-IR of male rats.

	FINS (*μ*U/mL)	HOMA-IR
Control group	5.42 ± 0.90	1.373 ± 0.028
Diabetic group	11.41 ± 2.13^##^	12.272 ± 0.331^##^
Metformin group	5.74 ± 0.88^*∗∗*^	3.138 ± 0.125^*∗∗*^
SGD group	6.36 ± 1.45^*∗∗*^	4.099 ± 0.180^*∗∗*^

Values are expressed as mean ± SD. ^##^*P* < 0.01 versus the control group; ^*∗∗*^*P* < 0.01 versus the diabetic group.

**Table 7:** Effect of the administration of saline (3 mL/Kg) or SGD (1240 mg/Kg) on TG, TC, HDL-C, LDL-C, GHb, and hepatic glycogen of male rats.

	TG (mmol/L)	TC (mmol/L)	HDL-C (mmol/L)	LDL-C (mmol/L)	GHb	Hepatic glycogen (mg/g tissue)
Control group	0.74 ± 0.18	1.99 ± 0.20	1.67 ± 0.37	0.67 ± 0.09	16.96 ± 1.49	5.34 ± 0.81
Diabetic group	1.58 ± 0.49^##^	3.03 ± 0.17^##^	0.62 ± 0.19^##^	1.14 ± 0.21^##^	24.53 ± 2.64^##^	3.87 ± 0.11^##^
Metformin group	0.84 ± 0.17^*∗∗*^	2.57 ± 0.26^*∗*^	0.74 ± 0.08	0.80 ± 0.17^*∗∗*^	20.58 ± 1.91^*∗*^	4.96 ± 1.12^*∗*^
SGD group	0.84 ± 0.41^*∗∗*^	2.58 ± 0.30^*∗*^	0.85 ± 0.18^*∗*^	0.88 ± 0.14^*∗*^	20.53 ± 3.85^*∗*^	5.28 ± 1.33^*∗*^

Values are expressed as mean ± SD. ^##^*P* < 0.01 versus the control group; ^*∗*^*P* < 0.05 versus the diabetic group; ^*∗∗*^*P* < 0.01 versus the diabetic group.

**Table 8:** SOD, CAT, and GSH-PX activities and MDA level of the control group, diabetic group, metformin group, and SGD group.

	SOD activity (U/ml)	MDA level (nmol/ml)	CAT activity (U/ml)	GSH-PX activity (U/ml)
Control group	449.33 ± 21.99	1.75 ± 0.13	13.87 ± 3.42	576.42 ± 13.37
Diabetic group	388.15 ± 20.22^##^	3.87 ± 0.52^##^	4.53 ± 1.91^##^	338.03 ± 84.16^##^
Metformin group	415.57 ± 16.23^*∗*^	2.49 ± 0.21^*∗∗*^	10.94 ± 2.92^*∗∗*^	521.89 ± 40.05^*∗∗*^
SGD group	414.68 ± 14.34^*∗*^	2.44 ± 0.61^*∗∗*^	10.52 ± 3.90^*∗∗*^	510.92 ± 36.34^*∗∗*^

Values are expressed as mean ± SD. ^##^*P* < 0.01 versus the control group; ^*∗*^*P* < 0.05 versus the diabetic group; ^*∗∗*^*P* < 0.01 versus the diabetic group.

**Table 9:** Related gene mRNA expression levels in rat livers.

	INSR	IRS-2	GLUT-4	PI3K	AKT
Control group	1.24 ± 0.31^*∗*^	1.31 ± 0.42^*∗*^	1.16 ± 0.36^*∗*^	1.25 ± 0.41^*∗*^	1.05 ± 0.21^*∗*^
Diabetic group	0.45 ± 0.02	0.63 ± 0.21	0.57 ± 0.25	0.59 ± 0.28	0.49 ± 0.13
Metformin group	0.98 ± 0.24^*∗*^	1.06 ± 0.32^*∗*^	0.93 ± 0.21^*∗*^	1.01 ± 0.26^*∗*^	0.93 ± 0.22^*∗*^
SGD group	0.85 ± 0.29^*∗*^	0.95 ± 0.37^*∗*^	0.89 ± 0.29^*∗*^	0.98 ± 0.28^*∗*^	0.88 ± 0.25^*∗*^

Values are expressed as mean ± SD. ^*∗*^*P* < 0.05 versus the diabetic group.
