# Corrigendum to “The Effect of Sanggua Drink Extract on Insulin Resistance through the PI3K/AKT Signaling Pathway”

**DOI:** 10.1155/2018/1404126

**Published:** 2018-08-19

**Authors:** Yu Cai, Ying Wang, Fei Zhi, Qi-Chang Xing, Yun-Zhong Chen

**Affiliations:** ^1^College of Pharmacy, Hubei University of Chinese Medicine, Wuhan 430065, China; ^2^Institute of Engineering Technology of Chinese Traditional Medicine and Health Food of Hubei Province, Wuhan 430065, China

In the article titled “The Effect of Sanggua Drink Extract on Insulin Resistance through the PI3K/AKT Signaling Pathway” [1], a grant number was missing. The Acknowledgments section should be updated as follows:

The authors are grateful to the National Natural Science Foundation of China (Grant no. 81673867) and the Major Projects of Technology Innovation of Hubei Provincial Science and Technology Department (Grant no. 2016ACA145) for the financial support.
